# Bacterial mutagenicity test data: collection by the task force of the Japan pharmaceutical manufacturers association

**DOI:** 10.1186/s41021-021-00206-1

**Published:** 2021-09-30

**Authors:** Atsushi Hakura, Takumi Awogi, Toshiyuki Shiragiku, Atsushi Ohigashi, Mika Yamamoto, Kayoko Kanasaki, Hiroaki Oka, Yasuaki Dewa, Shunsuke Ozawa, Kouji Sakamoto, Tatsuya Kato, Eiji Yamamura

**Affiliations:** 1 Global Drug Safety, Eisai Co., Ltd., 5–1-3 Tokodai, Tsukuba, Ibaraki 300–2635 Japan; 2 Manufacturing Process Development Department, Otsuka Pharmaceutical Co., Ltd., 224–18 Hiraishi-Ebisuno, Kawauchi-cho, Tokushima-shi, Tokushima, 771–0182 Japan; 3 Tokushima Research Institute, Otsuka Pharmaceutical Co., Ltd., 463–10 Kagasuno, Kawauchi-cho, Tokushima-shi, Tokushima, 771–0192 Japan; 4 Process Chemistry Labs, Astellas Pharma Inc., 160–2 Akahama, Takahagi, Ibaraki 318–0001 Japan; 5 Drug Safety Research Labs, Astellas Pharma Inc., 21 Miyukigaoka, Tsukuba, Ibaraki 305–8585 Japan; 6 Laboratory for Drug Discovery and Development, Shionogi & Co., Ltd., 3-1-1 Futaba-cho, Osaka, Toyonaka-shi 561-0825 Japan; 7 Toxicology Laboratory, Taiho pharmaceutical Co., Ltd., 224–2 Ebisuno, Hiraishi, Kawauchi-cho, Tokushima, 771–0194 Japan; 8 Toxicology Research Laboratory, Kyorin Pharmaceutical Co., Ltd., 1848 Nogi, Nogi-machi, Shimotsuga-gun, Tochigi, 329–0114 Japan; 9 Drug Safety, Taisho Pharmaceutical Co., Ltd., 1–403, Yoshino-cho, Kita-ku, Saitama-shi, 331–9530 Japan; 10 Safety Research Laboratories, Mitsubishi Tanabe Pharma Co., 2-2-50 Kawagishi, Toda-shi, Saitama, 335-8505 Japan

**Keywords:** Ames test, Mutagenicity, Bacteria, In silico, Structure-activity relationship, Derek Nexus, CASE Ultra

## Abstract

**Background:**

Ames test is used worldwide for detecting the bacterial mutagenicity of chemicals*.* In silico analyses of bacterial mutagenicity have recently gained acceptance by regulatory agencies; however, current in silico models for prediction remain to be improved. The Japan Pharmaceutical Manufacturers Association (JPMA) organized a task force in 2017 in which eight Japanese pharmaceutical companies had participated. The purpose of this task force was to disclose a piece of pharmaceutical companies’ proprietary Ames test data.

**Results:**

Ames test data for 99 chemicals of various chemical classes were collected for disclosure in this study. These chemicals are related to the manufacturing process of pharmaceutical drugs, including reagents, synthetic intermediates, and drug substances. The structure-activity (mutagenicity) relationships are discussed in relation to structural alerts for each chemical class. In addition, in silico analyses of these chemicals were conducted using a knowledge-based model of Derek Nexus (Derek) and a statistics-based model (GT1_BMUT module) of CASE Ultra. To calculate the effectiveness of these models, 89 chemicals for Derek and 54 chemicals for CASE Ultra were selected; major exclusions were the salt form of four chemicals that were tested both in the salt and free forms for both models, and 35 chemicals called “known” positives or negatives for CASE Ultra. For Derek, the sensitivity, specificity, and accuracy were 65% (15/23), 71% (47/66), and 70% (62/89), respectively. The sensitivity, specificity, and accuracy were 50% (6/12), 60% (25/42), and 57% (31/54) for CASE Ultra, respectively. The ratio of overall disagreement between the CASE Ultra “known” positives/negatives and the actual test results was 11% (4/35). In this study, 19 out of 28 mutagens (68%) were detected with TA100 and/or TA98, and 9 out of 28 mutagens (32%) were detected with either TA1535, TA1537, WP2*uvrA,* or their combination.

**Conclusion:**

The Ames test data presented here will help avoid duplicated Ames testing in some cases, support duplicate testing in other cases, improve in silico models, and enhance our understanding of the mechanisms of mutagenesis.

**Supplementary Information:**

The online version contains supplementary material available at 10.1186/s41021-021-00206-1.

## Introduction

The bacterial mutagenicity test, known as Ames test, is used worldwide to detect the mutagenicity of chemicals [1, 2]. Ames test is utilized not only for research purposes but also for submission to regulatory agencies for the approval of chemical substances [3, 4]. Recently, in silico evaluation of bacterial mutagenicity has been accepted by regulatory agencies [e.g.*,* the International Council for Harmonisation of Technical Requirements for Pharmaceuticals for Human Use (ICH) M7 guideline [5] for hazard identification of mutagenic impurities in medicinal drugs]. In recent years, several in silico models for predicting bacterial mutagenicity have been developed. However, the prediction level is not fully satisfactory and remains to be improved [6–8]. One way to improve this is to collect Ames test data, particularly for chemicals in some chemical classes where a limited number of test data are available.

For this reason, the Japan Pharmaceutical Manufacturers Association (JPMA) organized a task force for Ames data sharing. The purpose of this task force was to disclose a piece of pharmaceutical companies’ proprietary Ames test data to make them available to anyone for utilization in research or submission to regulatory agencies, and to improve in silico models by using them as training set examples. Eight Japanese pharmaceutical companies participated in this task force, and Ames test data for 99 chemicals were collected. These chemicals are related to the manufacturing process of pharmaceutical drugs, including reagents, synthetic intermediates, and drug substances. In addition, in silico analyses of these chemicals for bacterial mutagenicity were conducted using a knowledge-based model (Derek Nexus, Lhasa Limited) or a statistics-based model (CASE Ultra, MultiCASE Inc.).

In this report, we present the Ames test data and in silico predictions for 99 chemicals of various chemical classes and discuss their structure-activity relationships in relation to structural alerts for each chemical class.

## Materials and methods

### Materials

Ninety-nine chemicals were tested and collected by this task force. Table 1 lists the chemical identification (ID), chemical name, CAS registry number (CAS No.), source, purity of the test chemicals used, and test site. Table 2 lists the chemical ID, chemical name (arranged by chemical classes), chemical structure, solvent used to dissolve the test chemicals, summarized Ames test results, and in silico analyses. In this study, free and salt forms were treated as different chemicals.

**Table 1:** Chemical ID, test chemical, CAS No. source or supplier of test chemical, purity, and test site

Chemical ID	Test chemical	CAS No.	Source or supplier of test chemical	Purity (%)	Test site
1	1-Iodo-4-nitrobenzene	636–98-6	Maruzen Chemicals	99.9	CERI
2	2-Nitro-5-(1-piperazinyl)benzaldehyde HCl	13236300^*a*^	Otsuka Pharmaceutical	100	JISHA
3	Methyl 2-methyl-3-nitrobenzoate	59382–59-1	Otsuka Pharmaceutical	99.57	JISHA
4	2-Nitro-5-(1-piperazinyl)benzaldehyde dimethyl acetal	101291629^*a*^	Otsuka Pharmaceutical	99.8	JISHA
5	5-Chloro-2-nitrobenzaldehyde dimethyl acetal	13796–06-0	Otsuka Pharmaceutical	99.97	JISHA
6	2-Nitro-5-(1-piperazinyl)cinnamic acid	NR	Otsuka Pharmaceutical	99.8	JISHA
7	2-Fluoro-4-nitrophenol	403–19-0	Tokyo Chemical Industry	99.6	BoZo Research Center
8	3-Hydroxy-4-nitrobenzoic acid	619–14-7	Eisai	99.6	UBE
9	Pranidipine; Methyl (2*E*)-phenylprop-2-en-1-yl 2,6-dimethyl-4-(3-nitrophenyl)-1,4-dihydropyridine-3,5-dicarboxylate	99522–79-9	Otsuka Pharmaceutical	99.97	Otsuka Pharmaceutical
10	4-Amino-2-fluorophenol	399–96-2	Tokyo Chemical Industry	99.4	BoZo Research Center
11	Methyl 3-amino-2-methyl benzoate	18583–89-6	Otsuka Pharmaceutical	94.43	JISHA
12	Sodium 3-[2-amino-5-(1-piperazinyl)phenyl]propionate	101328646^*a*^	Otsuka Pharmaceutical	99.5	JISHA
13	Methyl 4-amino-2-methoxybenzoate	27492–84-8	Tokyo Chemical Industry	98.9	BoZo Research Center
14	Methyl 3-amino-4,6-dibromo-2-methylbenzoate	119916–05-1	Otsuka Pharmaceutical	98.74	JISHA
15	4-(2-Methoxy-phenyl)-thiazol-2-ylamine	93209–95-1	Shionogi	99.99	CMIC Pharma Science
16	4-Hexyl-1,3-thiazol-2-amine	90770–58-4	Shionogi	99.72	CMIC Pharma Science
17	2-Amino-4-hydroxythiazole	7146–26-1	Oakwood Products	98	LSI Medience
18	Thiazole-2,4-diamine	67355–26-4	Oxchem	98	LSI Medience
19	6-(2,3-Epoxypropoxy)-2(1*H*)-quinolinone	143343–78-6	Otsuka Pharmaceutical	94.44	JISHA
20	6-(4-(3,4-Dimethoxybenzoyl)-2,3-dihydroxypiperazin-1-yl)-3,4-dihydriquinolin-2(*1H*)-one	NR	Otsuka Pharmaceutical	98.12	Otsuka Pharmaceutical
21	8-Hydroxy-2(1*H*)-quinolinone	15450–76-7	Otsuka Pharmaceutical	99.55	JISHA
22	3,4-Dimethoxy-*N*-{2-[(2-oxo-1,2,3,4-tetrahydroquinolin-6-yl)amino]ethyl}benzamide	NR	Otsuka Pharmaceutical	99.96	Otsuka Pharmaceutical
23	6-(3-Oxopiperazin-1-yl)-3,4-dihydroquinolin-2(*1H*)-one	NR	Otsuka Pharmaceutical	99.56	Otsuka Pharmaceutical
24	3,4-Dihydro-5-(1-piperazinyl)-2-(1*H*) quinolinone	87154–95-8	Otsuka Pharmaceutical	> 99.9	JISHA
25	6-(4-(4-Hydroxy-3-methoxybenxoyl)piperazin-1-yl)-3,4-dihydroquinolin-2(*1H*)-one	NR	Otsuka Pharmaceutical	99.87	Otsuka Pharmaceutical
26	6-(1-Cyclohexyl-1*H*-tetrazol-5-yl)butoxy]-2(1*H*)-quinolinone	73963–62-9	Otsuka Pharmaceutical	100	Otsuka Pharmaceutical
27	*trans*-3,4-Dihydro-6-[4-[1-(4-hydroxycyclohexyl)-1*H*-tetrazol-5-yl]butoxy]-2(1*H*)-quinolinone	87153–04-6	Otsuka Pharmaceutical	99.93	Otsuka Pharmaceutical
28	Grepafloxacin; (*RS*)-1-Cyclopropyl-6-fluoro-5-methyl-7-(3-methylpiperazin-1-yl)-4-oxo-quinoline-3-carboxylic acid	119914–60-2	Otsuka Pharmaceutical	99.66	JISHA
29	Grepafloxacin HCl; (*RS*)-1-Cyclopropyl-6-fluoro-5-methyl-7-(3-methylpiperazin-1-yl)-4-oxo-quinoline-3-carboxylic acid monohydrochloride	161967–81–3	Otsuka Pharmaceutical	99.59	Otsuka Pharmaceutical
30	Ethyl 1-cyclopropyl-7-brorno-6-fluoro-1,4-dihydro-5-methyl-4-oxo-3-quinolinecarboxylate	119916–33-5	Otsuka Pharmaceutical	99.88	JISHA
31	2,4-Bis(trimethylsiloxy)-5-fluoropyrimidine	17242–85-2	Otsuka Pharmaceutical	99.3	JISHA
32	1,3-Dimethyl-2,4-pyrimidinedione	874–14-6	Otsuka Pharmaceutical	99.6	JISHA
33	1-(Ethoxymethyl)-5-fluoro-pyrimidine-2,4-dione	57610–22-7	Otsuka Pharmaceutical	99.7	JISHA
34	3-(1-Ethoxymethyl-5-fluoro-1,2,3,4-tetrahydro-2,4-dioxopyrimidin-3-yl)carbonylbenzoic acid	129971–17-1	Otsuka Pharmaceutical	99	JISHA
35	3-(3-Benzyloxycarbonylbenzoyl)-1-ethoxymethyl-5-fluoro-2,4-pyrimidinedione	NR	Otsuka Pharmaceutical	99.8	JISHA
36	1-Hydroxybenzotriazole hydrate	123333–53-9	Otsuka Chemical	99	BML
37	3*H*-[1,2,3]Triazolo[4,5-*b*]pyridin-3-ol	39968–33-7	Tokyo Chemical Industry	99	BML
38	1-[Bis (dimethylamino)methylene]-1*H*-1,2,3-triazolo[4,5-*b*]pyridinium 3-oxid hexafluorophosphate	148893–10-1	Sigma-Aldrich	99	BML
39	Methylcarbamoyl-phenyloxadiazole	1374817–07-8	Shionogi	98.77	CERI
40	4-(4,6-Dimethoxy-1,3,5-triazin-2-yl)-4-methylmorpholinium chloride *n*-hydrate	3945-69-5	Tokuyama	84.8	BML
41	*N*-Phenylbis(trifluoromethanesulfonimide)	37595–74-7	Tokyo Chemical Industry	99.9	SNBL
42	1,1,1-Trifluoro-*N*-phenylmethanesulfonamide	456–64-4	Tokyo Chemical Industry	99.9	SNBL
43	Perfluoro-1-butanesulfonyl fluoride	375–72-4	Funakoshi	> 90	BML
44	Diisopropyl sulfate	2973-10-6	Tokyo Chemical Industry	97	BML
45	Methyl *p*-toluenesulfonate	80–48-8	Kanto Chemical	98	BML
46	Ethyl trifluoromethanesulfonate	425–75-2	Tokyo Chemical Industry	99.8	SNBL
47	2-Nitrobenzenesulfonyl chloride	1694-92-4	Mitsubishi Tanabe Pharma	100.1	Koei Techno
48	*p*-Toluenesulfonyl chloride	98–59-9	Tokyo Chemical Industry	99	BML
49	4,6-Dibromo-3-fluoro-2-methylbenzoyl chloride	11916–28-8	Otsuka Pharmaceutical	99.18	JISHA
50	Benzyl 3-chloroformylbenzoate	67852–96-4	Otsuka Pharmaceutical	99.3	JISHA
51	3-(1-Ethoxymethyl-5-fluoro-1,2,3,4-tetrahydro-2,4-dioxopyrimidin-3-yl)carbonylbenzoyl chloride	1380098–51-0	Otsuka Pharmaceutical	91.1	JISHA
52	6-(3-Chloro-2-hydroxypropoxy)-2(1*H*)-quinolinone	128669–85-8	Otsuka Pharmaceutical	95.18	JISHA
53	Chloroacetonitrile	107–14-2	Tokyo Chemical Industry	99.9	LSI Medience
54	1-Bromohexane	111–25-1	Tokyo Chemical Industry	99.8	BSRC
55	2-Chloro-*N*-methoxy-*N*-methylacetamide	67442–07-3	Tokyo Chemical Industry	99.9	CMIC Pharma Science
56	Ethyl 5-chloro-2-[2-(trifluoromethyl)phenyl]pentanimidate HCl	1123197–78-3	Eisai	97.8	UBE
57	Liothyronine sodium	55–06-1	Acros Organics	95	Taisho
58	(4-Bromo-3,5-dimethoxyphenyl)methanol	61367–62-2	Eisai	100	UBE
59	Ethyl (4,6-dibromo-3-fluoro-2-methylbenzoyl)acetate	119916–30-2	Otsuka Pharmaceutical	98.88	JISHA
60	Catena-*m*-[2-ethoxycarbonyl-3-(4,6-dibromo-3-fluoro-2-methylphenyl)-3-oxidoacrylato(2-)-O,O′,O″,O″′]magnesium (II)	NR	Otsuka Pharmaceutical	87.2	JISHA
61	Methyl 4,6-dibromo-3-fluoro-2-methylbenzoate	119916–08-4	Otsuka Pharmaceutical	99.72	JISHA
62	4,6-Dibromo-3-fluoro-2-methylbenzoic acid	11916–27-7	Otsuka Pharmaceutical	98.79	JISHA
63	Sodium 4,6-dibromo-3-fluoro-2-methylbenzoate	NR	Otsuka Pharmaceutical	91.79	JISHA
64	Ethyl 2-(4,6-dibromo-3-fluoro-2-methyl benzoyl)-3-cyclopropylaminopropenoate	NR	Otsuka Pharmaceutical	99.95	JISHA
65	Ethyl 2-(4,6-dibromo-3-fluoro-2-methylbenzoyl)-3-ethoxypropenoate	NR	Otsuka Pharmaceutical	100	JISHA
66	Cinnamyl 3-aminocrotonate	113898–97-8	Otsuka Pharmaceutical	98.4	JISHA
67	Cinnamyl acetoacetate	57582–46-4	Otsuka Pharmaceutical	99.4	JISHA
68	Benzyl hydrogen isophthalate	113266–88-9	Otsuka Pharmaceutical	100	JISHA
69	Sodium benzyl isophthalate	NR	Otsuka Pharmaceutical	95.1	JISHA
70	Dibenzyl isophthalate	16034–14-3	Otsuka Pharmaceutical	99	JISHA
71	Diethyl phosphoryl chloride	814–49-3	Tokyo Chemical Industry	99	BML
72	Bis(diphenylphosphino)ferrocene	12150–46-8	Hokko Chemical	99.4	BML
73	Phosphorus (III) bromide	7789–60-8	Tokyo Chemical Industry	98	BML
74	Triethyl phosphonoacetate	867–13-0	Tokyo Chemical Industry	98.4	BML
75	Dicyclohexyl(2′,6′-dimethoxybiphenyl-2-yl)phosphine	657408–07-6	Johnson Matthey	100	BML
76	2-Dicyclohexylphosphino-2′,4′,6′-triisopropylbiphenyl (XPhos)	564483–18-7	Mitsubishi Tanabe Pharma	99.87	Koei Techno
77	Zinc cyanide	557–21-1	Alfa Aesar	98.9	BSRC
78	3-Cyano-2,6-dihydroxypyidine monosodium salt	91467–46-8	Otsuka Pharmaceutical	98.3	JISHA
79	3-Cyano-2,6-dihydroxypyridine	35441–10-2	Otsuka Pharmaceutical	99.8	JISHA
80	6-Benzoyloxy-3-cyano-2-hydroxypyridine	103941–70-4	Otsuka Pharmaceutical	100	JISHA
81	Ethyl oxoacetate	924–44-7	Weylchem	99.7	FDSC
82	2-Fluoro-3-hydroxy-5-methoxybenzaldehyde	883576–31-6	Eisai	99.3	UBE
83	4-Bromobenzaldehyde	1122-91-4	Tokyo Chemical Industry	99.9	FDSC
84	4-Pentyn-1-ol	5390-04-5	Avra Synthesis	97.85	LSI Medience
85	(*tert*-Butoxycarbonyl)hydrazide	870–46-2	Shanghai Unibest Biopharma	84.8	BML
86	4,6-Dibromo-3-methoxycarbonyl-2-methylbenzenediazonium tetrafluoroborate	NR	Otsuka Pharmaceutical	93.2	JISHA
87	9-Fluorenylmethyl alcohol	24324–17-2	Tokyo Chemical Industry	99.9	BSRC
88	*N*-(3-Dimethylaminopropyl)-*N*′-ethylcarbodiimide HCl	25952–53-8	Toyobo	99	BML
89	Benzamidoxime	613–92-3	Shionogi	> 98	Koei Techno
90	Carbethoxymethyl-dimethylsulfonium bromide	5187-82-6	Apollo Scientific	96.5^*b*^	FDSC
91	3,4-Dihydro-2*H*-pyran	110–87-2	Tokyo Chemical Industry	99	BML
92	(2*S*)-2-[(*tert*-Butoxycarbonyl)amino]hexanedioic acid dimethyl ester	615258–01-0	Eisai	96.9	UBE
93	*tert*-Butyl 2-acryloylhydrazine-1-carboxylate	28689–14-7	Eisai	99.9	UBE
94	[4-(Hydroxymethyl)-2,6-dimethoxyphenyl]boronic acid	332394–37-3	Eisai	99.9	UBE
95	Triethylsilane	617–86-7	Tokyo Chemical Industry	> 98	BML
96	1,3-Butanediol	107–88-0	Daicel	99.8	Nihon Bioresearch
97	Ammonium acetate	631–61-8	Wako Pure Chemical Industries	100	CERI
98	*p*-Toluenesulfinic acid sodium salt	7257-26-3	Tokyo Chemical Industry	99.7	BML
99	2,2,6,6-Tetramethylpiperidine 1-oxyl (free radical)	2564-83-2	Tokyo Chemical Industry	99.7	SNBL

*BSRC* Biosafety Research Center, Foods, Drug and Pesticides, *CERI* Chemicals Evaluation and Research Institute, *FDSC* Hatano Research Institute, Food and Drug Safety Center, *JISHA* Japan Industrial Safety and Health Association, *SNBL* Shin Nippon Biomedical Laboratories, *UBE* UBE Scientific Analysis Laboratory, *NR* not registered

^*a*^PubChem Compound ID

^*b*^purified after purchase

**Table 2:**
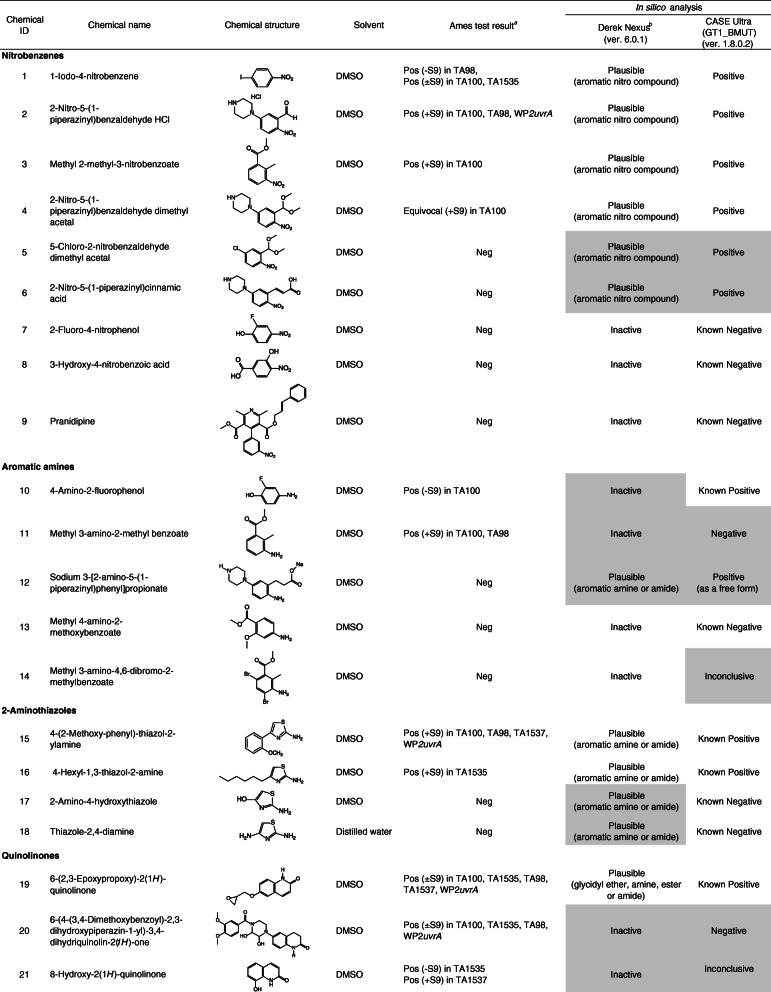

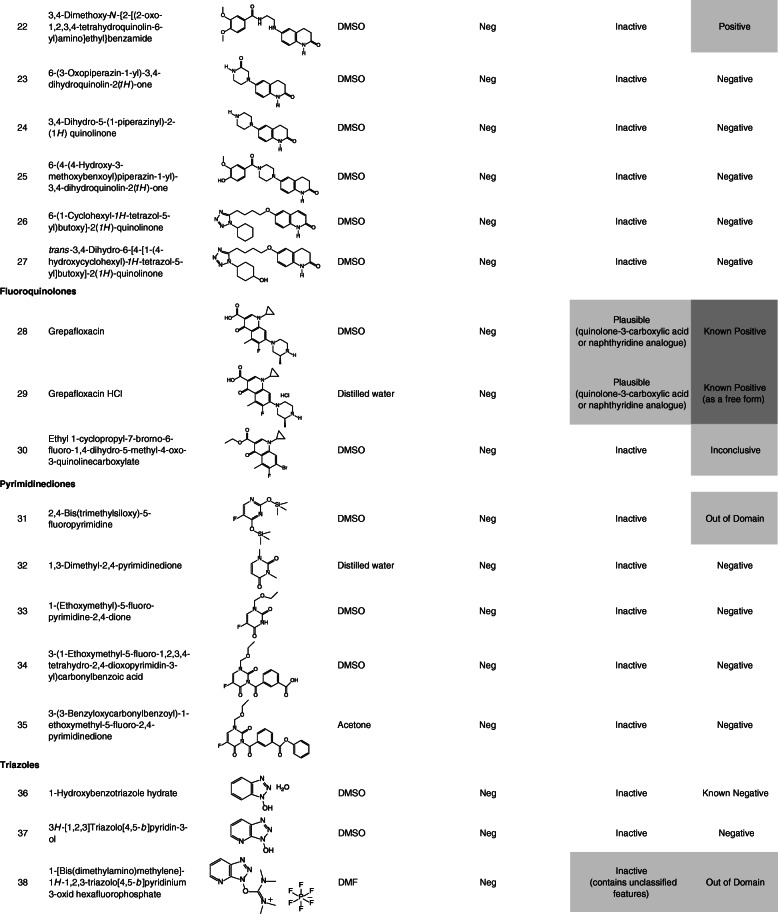

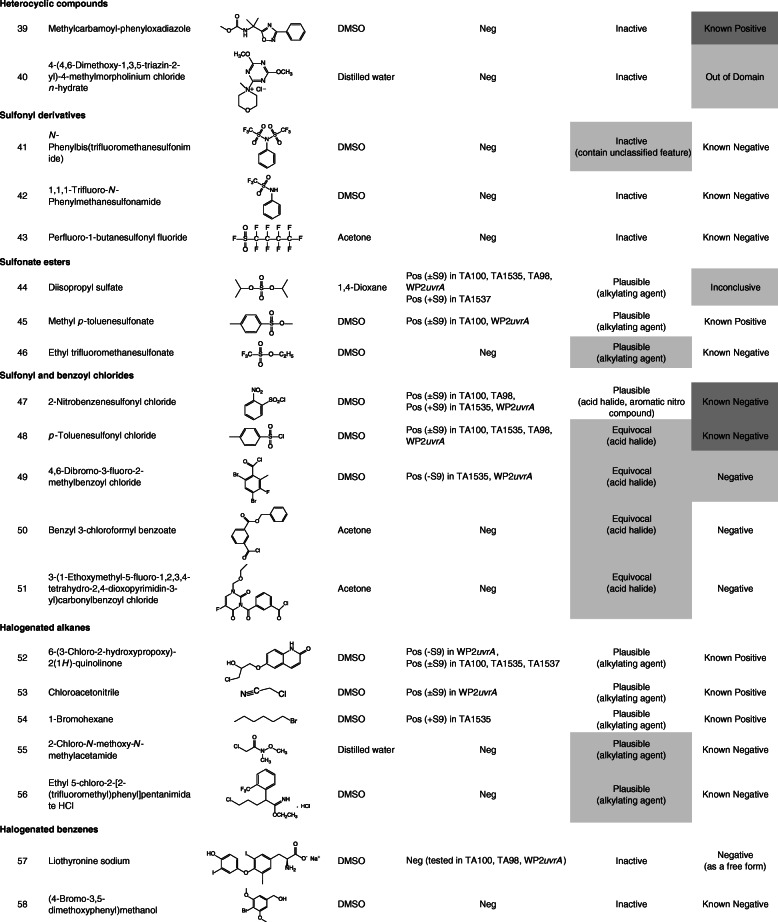

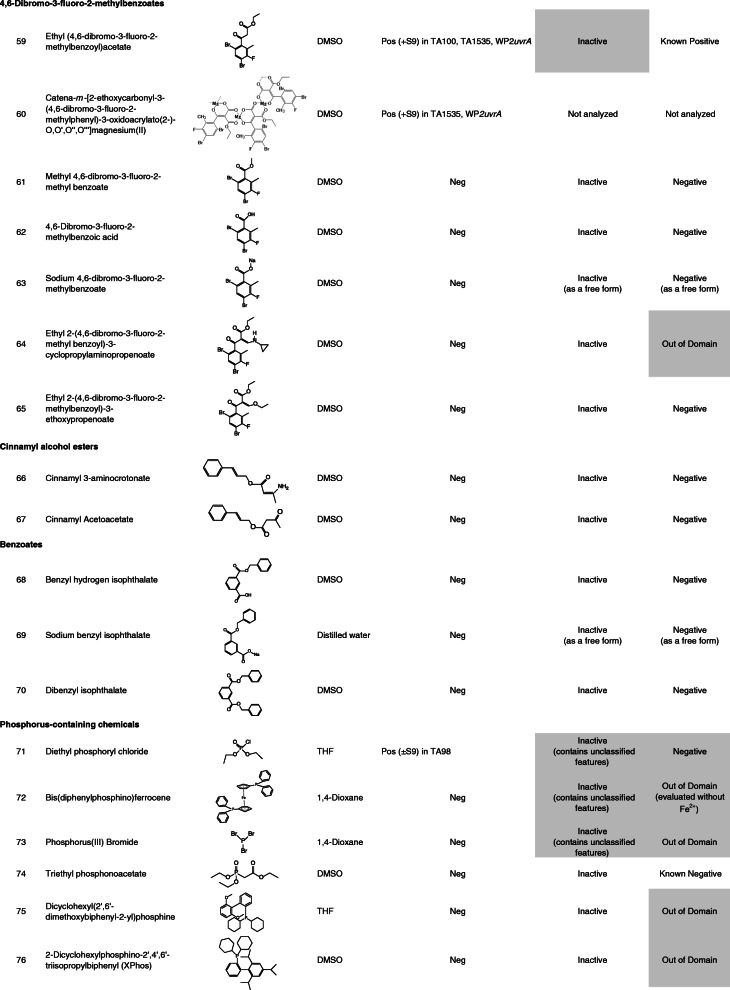

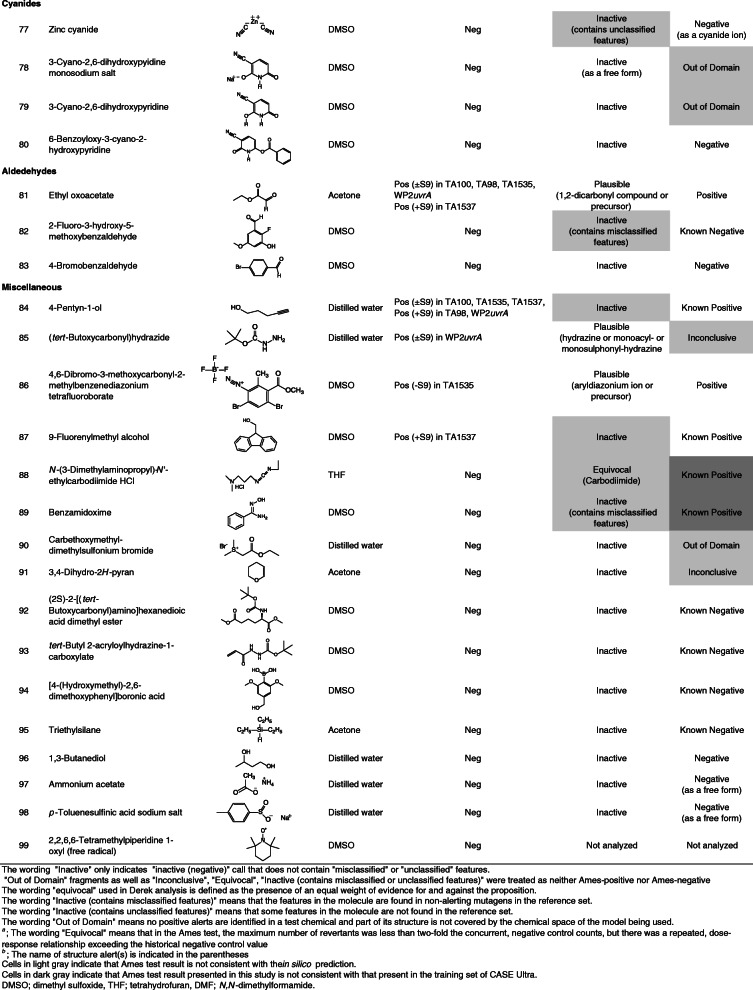
Chemical ID, chemical name, chemical structure, solvent used, Ames test result, and in silico analysis

S9 fraction, prepared from the liver of phenobarbital/5,6-benzoflavone-pretreated male Sprague-Dawley rats, was purchased from Oriental Yeast (Tokyo, Japan) or Kikkoman Biochemifa (Chiba, Japan). The S9 mix consisted of 10% (*v/v*) S9 fraction (approximately 1.0 mg protein/plate), 8 mM MgCl_2_, 33 mM KCl, 5 mM glucose-6-phosphate, 4 mM NADPH, 4 mM NADH, and 100 mM sodium phosphate (pH 7.4).

### Bacterial strains

Four strains of *Salmonella typhimurium,* namely TA100, TA1535, TA98, and TA1537, and one strain of *Escherichia coli,* either WP2*uvrA* or WP2*uvrA*/pKM101 (for chemical IDs 21, 56, 58, 82, 93, and 94), were used in each Ames test. Chemical ID 57 was tested using only TA100, TA98, and WP*2uvrA*. These tester strains are recommended for use in bacterial mutagenicity test by the Organisation for Economic Cooperation and Development (OECD) test guideline 471 [3].

### Ames test

All Ames tests were conducted using the preincubation method [9, 10]. Briefly, frozen stock cultures of each strain were inoculated into a conical flask or L-tube containing nutrient broth medium (2.5% w/v; Oxoid Nutrient Broth No.2, Hampshire, UK), and then cultured in a shaking incubator at 37 °C to obtain bacterial cells in the early stationary phase. The cell density of each culture was confirmed to be > 1 × 10^9^ cells/mL. For the tests carried out in the absence of S9 mix, 0.1 mL of the negative (vehicle) control solution, test chemical solution at various concentrations, or positive control solution was added to a test tube, to which 0.5 mL of 100 mM sodium phosphate buffer (pH 7.4) and 0.1 mL of bacterial culture were added. For the tests carried out in the presence of S9 mix, S9 mix was added in place of phosphate buffer. After mixing, the test tubes were preincubated at 37 °C for 20 min in a shaking water bath. After completion of the preincubation, the treatment mixture was immediately added and mixed with 2 mL of 0.05 mM L-histidine/0.05 mM D-biotin molten top agar (for *Salmonella* strains) or 0.05 mM L-tryptophan (for *E. coli* strains), and the content was poured onto a plate of minimal-glucose agar medium. The plates were incubated at 37 °C for approximately 48 h, and the revertant colonies that appeared were counted. The sign of bacterial background lawn was examined as an indicator of cytotoxicity. In addition, the presence or absence of a precipitate of the test chemical was checked. When acetone, tetrahydrofuran, *N,N*-dimethylformamide, or 1,4-dioxane was used as the solvent, 0.05 mL of the vehicle was added to the test tube.

Multiple tests (dose-finding test, main test, or confirmatory test) were conducted for 86 chemicals. For 13 chemicals, a single test was conducted, and a clear conclusion was drawn. All tests were carried out in duplicate (two plates per dose) or triplicate (three plates per dose), except for chemical ID 96 in dose-finding tests (single plate per dose). All solvents used were of high purity and were appropriate for use in Ames test.

Ames test data were generated in-house or in several Japanese contract research organizations in compliance with the Good Laboratory Practice (GLP), except for chemical IDs 47 and 57 (Table 1, Supplementary Tables).

Mutagenicity was evaluated according to the so-called “two-fold” rule [11]. The test chemical was judged to be positive (mutagenic) if the following criteria were satisfied: (1) the maximum number of revertants was two-fold or more relative to the negative (vehicle) control, (2) a dose-dependent increase in the number of revertants was observed, and (3) the results were reproducible between each test (if tests were conducted twice or thrice). Historical negative control counts in each laboratory were also considered for evaluation. Only chemical ID 4 was judged to be equivocal; although there was a clear dose-response relationship with reproducibility, the maximum number of revertants exceeded the upper limit of the historical negative control range, which was less than two-fold higher than the concurrent negative control counts.

### In silico analyses

Chemicals were analyzed using a knowledge-based model [Derek Nexus (Derek), ver. 6.0.1; Lhasa Limited, Leeds, UK] and a statistics-based model (CASE Ultra, GT1_BMUT, ver. 1.8.0.2; MultiCASE Inc., OH, USA).

## Results and discussion

The data for 99 chemicals, including four chemicals in the free and salt forms (chemical IDs 28 and 29, 62 and 63, 68 and 69, 78, and 79, respectively), were collected by the task force. The four pairs of these chemicals showed the same (negative) result with a similar toxicity between each pair, except for a pair of chemical IDs 28 and 29. Individual data are shown in Supplementary Tables. Table 2 lists the summarized Ames test and in silico analysis data of the test chemicals, which were arranged according to chemical classes. One-third of these chemicals were included in the training set for the latest version of CASE Ultra (where chemicals are presented as “Known positive” or “Known negative” in Table 2). The test chemicals were classified into the following chemical classes: nitrobenzenes, aromatic amines, 2-aminothiazoles, quinolinones, fluoroquinolones, pyrimidinediones, triazoles, heterocyclic compounds, sulfonyl derivatives, sulfonate esters, sulfonyl and benzoyl chlorides, halogenated alkanes, halogenated benzenes, 4,6-dibromo-3-fluoro-2-methylbenzoates, cinnamyl alcohol esters, benzoates, phosphorus-containing compounds, cyanides, aldehydes, and miscellaneous.

### Structure-activity relationships

Although some chemical classes have only a few chemicals, we discuss the structure-activity (mutagenicity) relationships in relation to structural alerts.

#### Nitrobenzenes

The structure of nitroarenes is a representative alert for mutagenicity, although the simplest nitroarene nitrobenzene itself is not mutagenic [12–16]. All Ames-positive nitrobenzene derivatives were predicted to be mutagenic by both in silico models; however, in the present study, approximately half of the nitrobenzenes (5/9 chemicals) were non-mutagenic. The mutagenicity of nitroarenes can be generated through the reduction of the nitro moiety to the corresponding *N*-hydroxylamines by bacterial nitroreductase, and therefore can be efficiently detected in the absence of S9 mix [12–16]. Interestingly, chemical IDs 2–4 were mutagenic or equivocal only in the presence of S9 mix. One possible reason for nitrobenzene mutagenesis is the nitroreduction inside bacterial cells after oxidative metabolism in the S9 mix [15, 16].

#### Aromatic amines

The structure of aromatic amines is also a representative indicator of mutagenicity [12–14]. The primary mechanism of mutagenicity by aromatic amines is known to be the production of *N*-hydroxylations, typically by the CYP 1A2 enzyme, followed by *O*-esterification with acetate or sulfate [17, 18]. In this study, several aromatic amines (3/5 chemicals) were not mutagenic. Some substituents that generate electronic and/or steric effects probably inhibit mutagenicity through inhibition of drug-metabolizing enzymes involved and/or decreased stability of the nitrenium ion intermediate that was generated through cleavage of the N-O bond of esterified *N*-hydroxylamines and form adducts with DNA, leading to mutations [18, 19]. The mutagenicity of chemical ID 10 is probably due to reactive *para-*iminoquinone, which does not require metabolic enzymes.

#### 2-Aminothiazoles

The 2-aminothiazoles tested, which were five-membered aromatic amines containing hetero atoms of sulfur in position 1 and nitrogen in position 3, were half mutagenic (2/4 chemicals) and half non-mutagenic (2/4 chemicals), with a diverse substituent at position 4. 2-Aminothiazoles were all predicted to be mutagenic (as “Plausible” by Derek) through identification of the structural alerts of aromatic amines or amides. 2-Aminothiazole is mutagenic, and the mutagenicity of 2-aminothiazoles is induced via the formation of reactive nitrenium ion intermediates, such as aromatic amines [19–21]. The presence of a substituent at position 4 may enhance or reduce the mutagenicity of 2-aminothiazole.

#### Quinolinones

The six quinolinone derivatives (chemical IDs 22–27) were non-mutagenic, whereas the other three were mutagenic. The quinolinone structure was not an alert, as shown by both in silico models. Chemical ID 19 was mutagenic, probably because of the presence of epoxide. The mutagenicity of chemical ID 20 may be derived from the dihydroxylated piperazine moiety. Chemical ID 21, an 8-hydroxy derivative of quinolinone, was mutagenic only in TA1535, and TA1537, which shows a small number of negative control counts and is empirically known to be sensitive to some structures.

#### Fluoroquinolones

The mutagenicity of fluoroquinolones was dependent on WP2*uvrA*, WP2*uvrA*/pKM101, or TA102, which have an AT base pair at the primary reversion site [1–3]. Fluoroquinolone antibiotics, including grepafloxacin, were reported to be mutagenic in TA102 [22] and WP2*uvrA*/pKM101 [23], and the positive result was used as a training set in CASE Ultra. However, in this study, where WP2*uvrA* was used, the three fluoroquinolone derivatives, including grepafloxacin (chemical ID 28) and grepafloxacin HCl (chemical ID 29), were all non-mutagenic.

The difference of cytotoxicity (reduction in bacterial background lawn) in the two forms (chemical IDs 28 and 29) was much more than would be expected by normal variation. It may be worth looking at the role of the different solvents, including water and DMSO.

#### Pyrimidinediones

The five pyrimidinedione derivatives were all non-mutagenic. Both in silico models predicted these chemicals to be inactive/negative except for one chemical called the “Out of Domain” owing to the presence of two trimethylsilyl moieties, as shown by CASE Ultra. The structure of pyrimidinedione should not be an alert for mutagenicity.

#### Triazoles

All three triazole derivatives were non-mutagenic. Both in silico models predicted that these chemicals were inactive/negative except for the “Inactive containing unclassified features” and “Out of Domain” owing to the presence of a tertiary amine moiety, as shown by Derek and CASE Ultra, respectively. The structure of triazole is unlikely to be an indicator of mutagenicity.

#### Heterocyclic compounds

The two heterocyclic compounds, derivatives of oxadiazole (chemical ID 39) and 1,3,5-triazine (chemical ID 40), were both non-mutagenic. The finding that chemical ID 39 was non-mutagenic was not consistent with the “known positive” from CASE Ultra.

#### Sulfonyl derivatives

The three sulfonyl derivatives were all non-mutagenic, which was consistent with that in both in silico models, although Derek identified an unclassified feature of sulfonimide in chemical ID 41. The structure of the sulfonyl moiety is not an alert for mutagenicity.

#### Sulfonate esters

Chemical IDs 44 and 45 were both mutagenic, and this result was consistent with the results of both in silico models. Several sulfonate esters are well-known to be alkylating mutagens, and predicted as “plausible” mutagens by Derek. However, chemical ID 46 was not mutagenic. The mutagenic potency of sulfonates is dependent on both the leaving group and alkylsulfonate moiety, affecting their chemical reaction rate [24, 25] and chemoselectivity [26, 27]. A probable reason for them being non-mutagenic is the rapid hydrolysis (instability) of ethyl trifluoromethanesulfonate [28]. The alertness of some sulfonate esters can be improved by incorporating the chemical properties.

#### Sulfonyl and benzoyl chlorides

The two sulfonyl chlorides (chemical IDs 47 and 48) and benzoyl chloride (chemical ID 49) were mutagenic in the presence or absence of S9 mix. Dimethyl sulfoxide (DMSO) was used as the solvent. It was reported that when DMSO was used to dissolve sulfonyl chlorides or acyl chlorides (including benzoyl chlorides), these chemicals showed mutagenicity (or false positive results) due to the generation of mutagenic impurity (chlorodimethyl sulfide) in the test chemical formulations, with a few exceptions [29, 30]. Derek predicted sulfonyl and benzoyl chlorides to be equivocal, the definition of which is that there is evidence for and against being mutagenic. These chemicals may not be mutagenic with organic solvents other than DMSO, such as acetone, where sulfonyl and acyl chlorides are stable. Water is probably not appropriate as a solvent, because these chemicals are generally unstable. Further tests on chemical IDs 47–49 are necessary to draw the correct conclusions. Nevertheless, the data presented here may be valuable as data examples when using solvents inappropriate for this chemical class. The other two benzoyl chlorides, chemical IDs 50 and 51, were correctly judged to be non-mutagenic and dissolved in acetone.

#### Halogenated alkanes

Halogenated alkanes (halogen atoms excluding fluorine) can be alkylating mutagens without requiring metabolic activation. Similar to that of sulfonate esters, their mutagenic activity is dependent on the alkyl moieties and the leaving group of halogen ions. A possible reason why chemical IDs 55 and 56 were non-mutagenic is that the DNA adduct was not formed via inhibition of the SN_2_ reaction through steric hindrance by the bulky substituent around the carbon center adjacent to the chlorine atom. In this study, chemical ID 54 with a long alkyl chain (hexyl moiety) and a leaving group of bromine ions is marginally positive only in TA1535, which shows a low number of negative control counts in the presence of S9 mix, although *n*-butyl chloride with a shorter alkyl moiety is reported to be non-mutagenic [31]. Primary alkyl bromides with chains longer than the hexyl moiety are probably non-mutagenic.

#### Halogenated benzenes

The two halogenated benzenes were non-mutagenic. Chemical ID 57 was tested with three test strains, TA100, TA98, and WP2*uvrA*; the strains TA100 and TA98 were most sensitive among the five strains that are recommended for use by OECD test guideline 471 [3]. Halogenated benzenes are unlikely to be structural alerts for mutagenicity, as supported by Derek.

#### 4,6-Dibromo-3-fluoro-2-methylbenzoates

Five 4,6-dibromo-3-fluoro-2-methylbenzoate derivatives (chemical IDs 61 to 65) were non-mutagenic, and Derek and CASE Ultra did not show alerts for this structure. Therefore, the structure of 4,6-dibromo-3-fluoro-2-methylbenzoate is not an indicator of mutagenicity. The mutagenicity of chemical ID 59 might involve the enol (tautomerized) form of the 1,3-diketone moiety, followed by epoxidation of the double bond by the drug-metabolizing enzyme in S9 mix. The substitution at position 2 of the 1,3-diketone moiety may inhibit tautomerization, but not lead to the induction of mutagenicity (chemical IDs 64, 65). It remains unclear why chemical ID 60 was mutagenic. Mutagenicity may be associated with the magnesium-oxygen complex.

#### Cinnamyl alcohol esters

Both cinnamyl esters were non-mutagenic, as predicted by both in silico models. A double bond conjugated with a benzene ring is unlikely to be a structural indicator of mutagenicity.

#### Benzoates

All benzoates were non-mutagenic, as predicted by both in silico models.

#### Phosphorus-containing chemicals

Phosphorus-containing chemicals were all non-mutagenic except for chemical ID 71, which is electrophilic and routinely used in organic synthesis for the phosphorylation of amines [32]. For many of the phosphorus-containing chemicals tested, neither of the in silico models were able to make a definite, positive/negative prediction; the reference to negative by Derek contained unclassified features, and CASE Ultra called “Out of Domain”. This indicates that phosphorus-containing chemicals are outside the applicability domain because of the limited number of training set examples for each in silico model.

#### Cyanides

Cyanide ion (Chemical ID 77) and all the cyanide derivatives substituted with an aromatic ring were non-mutagenic. The cyanide moiety is unlikely to be a structural alert for mutagenicity, as supported by Derek.

#### Aldehydes

Chemical ID 81, an aldehyde conjugated with a single carbonyl moiety, was mutagenic, as predicted by both in silico models. The chemical properties of aldehydes largely differ between aliphatic and aromatic compounds; generally, the former is chemically reactive, whereas the latter is stable. Both aromatic aldehydes (chemical IDs 82 and 83) were non-mutagenic, which can be explained by the extremely low chemical reactivity of aromatic aldehydes.

#### Miscellaneous

The miscellaneous group consists of chemicals that cannot be simply classified into the above chemical classes. Many of the chemicals tested were non-mutagenic. Chemical ID 84 was mutagenic in the presence and absence of S9 mix, although there were no structural alerts identified by Derek. The cause of the mutagenicity is unclear, but aldehyde might be involved in the induction of mutagenicity, which may be generated from alcohol by the alcohol dehydrogenase present in bacteria [33]. The three chemicals (chemical IDs 85–87) were mutagenic. Chemical IDs 85 and 86 were mutagenic only in WP2*uvrA* and TA1535, respectively. Both chemicals were predicted to be mutagenic (Derek; Plausible, CASE Ultra; Inconclusive or Positive) by both in silico models. Chemical ID 87 was only mutagenic in TA1537, which would be a tester strain sensitive to some chemical structures, with a small number of negative control counts.

### In silico analyses

To calculate the sensitivity, specificity, and accuracy of in silico predictions, ten chemicals (chemical IDs 29, 47–49, 57, 60, 63, 69, 78, and 99) were excluded. Four chemicals tested in both forms were used for calculation in the free form (chemical IDs 28, 62, 68, and 79), but not in the salt form (chemical IDs 29, 63, 69, and 78). Chemical IDs 47–49 were false positive because probable inappropriate solvents were used. Chemical ID 57 was tested in only three strains (TA100, TA98 and WP2*uvrA*). For chemical IDs 60 and 99, the in silico models could not reach a conclusion because the former is a complex molecule and the latter is a radical. We treated “Out of Domain” fragments as well as “Inconclusive”, “Equivocal”, “Inactive (contains misclassified or unclassified features)”, as neither Ames-positive nor Ames-negative in this study.

In silico analysis using Derek (ver. 6.0.1) revealed the sensitivity, specificity, and accuracy to be 65% (15/23), 71% (47/66), and 70% (62/89), respectively. In contrast, in silico analysis using CASE Ultra (GT1_BMUT, ver. 1.8.0.2) revealed the sensitivity, specificity, and accuracy to be 50% (6/12), 60% (25/42), and 57% (31/54), respectively. Thus, Derek outperformed CASE Ultra (GT1_BMUT) in the predictive level of bacterial mutagenicity for all the parameters in this study, where the limited number of chemicals were compared.

Derek and Case Ultra occasionally called “inactive containing misclassified or unclassified features” (8 chemicals), and “Out of Domain” fragments (10 chemicals), respectively, indicating the need to expand the training or reference set for each in silico model to improve.

It is worth noting that when considering the performance of the in silico models, it is important to account for the ICH M7 approach of combining two complementary systems and an expert review to take a final decision rather than considering them separately [5, 34].

### Inconsistency with training set examples

The 35 chemicals (15 “known” positives and 20 “known” negatives) were part of the training set for CASE Ultra. The results for 4 of 35 chemicals (11%) did not agree with the known response for those chemicals in that training set. The four chemicals (chemical IDs 28, 39, 88, and 89) were non-mutagenic but were registered as mutagens in the training set for CASE Ultra. This disagreement ratio (11%) was in the same range as the Ames test non-reproducibility, identified by Piegorsch and Zeiger, who reported a value of approximately 13% [35]. The reasons why the Ames test evaluations did not match were mainly some differences in the test conditions (e.g.*,* plate-incorporation method vs. preincubation method, the type of strains used, source of test strains, preparation of overnight culture), and evaluation criteria (e.g.*,* two-fold rule vs. statistical analysis), and quality of test substances [10, 11, 36].

Two chemicals (chemical IDs 47 and 48) were mutagenic but were registered as non-mutagens in the CASE Ultra training set. This is probably because the solvent used in our study was not appropriate, as previously stated (see the section of “Sulfonyl and benzoyl chlorides” in the Structure-activity relationships section. Our data, together with individual data (Supplementary Tables), provide additional information and will help in reevaluating the Ames test data.

### Test strains to detect bacterial mutagens

In this study, 28 chemicals, including three sulfonyl and benzoyl chlorides (chemical IDs 47 to 49) were mutagenic. Among them, three chemicals (chemical IDs 16, 54, and 86), two chemicals (chemical IDs 21 and 87), two chemicals (chemical IDs 53 and 85), and two chemicals (chemical IDs 49 and 60), respectively, were only detected for mutagenicity in either TA1535, TA1537, WP2*uvrA*, or both TA1535 and WP2*uvrA*. Williams et al. [36] reported that 93% of bacterial mutagens can be detected with a combination of TA100 and TA98. However, the data of the present study show that only 19 out of 28 chemicals (68%) were detected either by TA100 or TA98. Therefore, the test strains TA1535, TA1537, and WP2*uvrA* may be useful for the efficient detection of bacterial mutagenicity.

## Conclusion

Ames test data were presented for 99 chemicals from eight pharmaceutical companies through the activity of the Ames data sharing task force. The chemicals were related to the manufacturing process of pharmaceutical drugs, including reagents, synthetic intermediates, and drug substances. The Ames test data presented herein will contribute to avoiding duplicated Ames test in some cases, supporting duplicate testing in other cases, improving in silico models, and enhancing our understanding of the mechanisms of mutagenesis.

## Supplementary Information

**Additional file 1.**

## Data Availability

All Ames data are available in the Supplementary Tables. Materials are not applicable.

## Abbreviations

9AA: 9-aminoacridine
2AA: 2-aminoanthracene
B[*a*]P: Benzo[*a*]pyrene
ICR-191: 6-chloro-9-[3-(2-chloroethylamino)propylamino]-2-methoxyacridine dihydrochloride
DMSO: Dimethyl sulfoxide
AF-2: 2-(2-furyl)-3-(5-nitro-2-furyl)​acrylamide
GLP: Good Laboratory Practice
ICH: International Council for Harmonisation of Technical Requirements for Pharmaceuticals for Human Use
JPMA: Japan Pharmaceutical Manufacturers Association
OECD: Organisation for Economic Cooperation and Development

## References

[1] Maron DM, Ames BN (1983). Revised methods for the *Salmonella* mutagenicity test. Mutat Res.

[2] Mortelmans K, Zeiger E (2000). The Ames *Salmonella*/microsome mutagenicity assay. Mutat Res.

[3] OECD (2020). Guideline for the Testing of Chemicals: Bacterial Reverse Mutation Test No. 471 OECD Environment, Health and Safety Publications Series on Testing and Assessment Organization for Economic Cooperation and Development, Paris.

[4] ICH Harmonized Tripartite Guideline, “Guidance on Genotoxicity testing and data interpretation for pharmaceuticals intended for human use”, S2 (R1), current step 4 version, 2011. https://database.ich.org/sites/default/files/S2_R1_Guideline.pdf.

[5] Harmonized Tripartite Guideline ICH (2017). “Assessment and control of DNA reactive (mutagenic) impurities in pharmaceuticals to limit potential carcinogenic risk”, M7 (R1), current step 4 version.

[6] Landry C, Kim MT, Kruhlak NL, Cross KP, Saiakhov R, Chakravarti S, Stavitskaya L (2019). Transitioning to composite bacterial mutagenicity models in ICH M7 (Q) SAR analyses. Regul Toxicol Pharmacol.

[7] Benigni R, Bossa C (2019). Data-based review of QSARs for predicting genotoxicity: the state of the art. Mutagenesis..

[8] Honma M. An assessment of mutagenicity of chemical substances by (quantitative) structure-activity relationship. Genes Environ. 2020;42(1):23. 10.1186/s41021-020-00163-1. 10.1186/s41021-020-00163-1 PMC7330942 32626544

[9] Kato M, Sugiyama K, Fukushima T, Miura Y, Awogi T, Hikosaka S, Kawakami K, Nakajima M, Nakamura M, Sui H, Watanabe K, Hakura A (2018). Negative and positive control ranges in the bacterial reverse mutation test: JEMS/BMS collaborative study. Genes Environ.

[10] Levy DD, Hakura A, Elespuru RK, Escobar PA, Kato M, Lott J, Moore MM, Sugiyama K (2019). Demonstrating laboratory proficiency in bacterial mutagenicity assays for regulatory submission. Mutat Res.

[11] Levy DD, Zeiger E, Escobar PA, Hakura A, van der Leede BM, Kato M, Moore MM, Sugiyama K (2019). Recommended criteria for the evaluation of bacterial mutagenicity data (Ames test). Mutat Res.

[12] Kazius J, McGuire R, Bursi R (2005). Derivation and validation of toxicophores for mutagenicity prediction. J Med Chem.

[13] Benigni R (2005). Structure-activity relationship studies of chemical mutagens and carcinogens: mechanistic investigations and prediction approaches. Chem Rev.

[14] Benigni R, Bossa C (2011). Mechanisms of chemical carcinogenicity and mutagenicity: a review with implications for predictive toxicology. Chem Rev.

[15] Shimizu M, Yano E (1986). Mutagenicity of mono-nitrobenzene derivatives in the Ames test and rec assay. Mutat Res.

[16] Suzuki J, Takahashi N, Kobayashi Y, Miyamae R, Ohsawa M, Suzuki S (1987). Dependence on *Salmonella typhimurium* enzymes of mutagenicities of nitrobenzene and its derivatives in the presence of rat-liver S9 and norharman. Mutat Res.

[17] Kim D, Guengerich FP (2005). Cytochrome P450 activation of arylamines and heterocyclic amines. Annu Rev Pharmacol.

[18] Gadaleta D, Manganelli S, Manganaro A, Porta N, Benfenati E (2016). A knowledge-based expert rule system for predicting mutagenicity (Ames test) of aromatic amines and azo compounds. Toxicology.

[19] Bentzien J, Hickey ER, Kemper RA, Brewer ML, Jane D, Dyekjær JD, East SP, Whittaker M (2010). An in silico method for predicting Ames activities of primary aromatic amines by calculating the stabilities of nitrenium ions. J Chem Inf Model.

[20] McCarren P, Bebernitz GR, Gedeck P, Glowienke S, Grondine MS, Kirman LC, Klickstein J, Schuster HF, Whitehead L (2011). Avoidance of the Ames test liability for aryl-amines via computation. Bioorg Med Chem.

[21] Seifried HE, Seifried RM, Clarke JJ, Junghans TB, San RHC (2006). A compilation of two decades of mutagenicity test results with the Ames *Salmonella typhimurium* and L5178Y mouse lymphoma cell mutation assays. Chem Res Toxicol.

[22] Yim G, McClure J, Surette MG, Davies JE (2011). Modulation of Salmonella gene expression by subinhibitory concentrations of quinolones. J Antibiotics.

[23] Hayasaki Y, Itoh S, Kato M, Furuhama K (2006). Mutagenesis induced by 12 quinolone antibacterial agents in Escherichia coli WP2uvrA/pKM101. Toxicol in Vitro.

[24] Hakura A, Mizuno Y, Goto M, Kawazoe Y (1986). Studies on chemical carcinogens and mutagens. XXXV. Standardization of mutagenic capacities of several common alkylating agents based on the concentration-time integrated dose. Chem Pharm Bull.

[25] Hakura A, Kawazoe Y (1986). Studies on chemical carcinogens and mutagens. XXXVI. Apparent activation energy for mutagenic modification induced in *E. coli* by alkylating agents. Estimation from mutation frequency. Chem Pharm Bull.

[26] Kawazoe Y, Tamura N, Yoshimura T (1982). Studies on chemical carcinogens. XXIII. A simple method for characterization of the alkylating ability of compounds by using 4-(*p*-nitrobenzyl)pyridine. Chem Pharm Bull.

[27] Snodin DJ (2006). Residues of genotoxic alkyl mesylates in mesylate salt drug substances: real or imaginary problems?. Regul Toxicol Pharmacol.

[28] Streitwieser A, Wilkins C, Kiehlmann E (1968). Kinetics and isotope effects in solvolyses of ethyl trifluoromethanesulfonate. J Am Chem Soc.

[29] Amberg A, Harvey JS, Czich A, Spirkl H-P, Robinson S, White A, Elder DP (2015). Do carboxylic/sulfonic acid halides really present a mutagenic and carcinogenic risk as impurities in final drug products?. Org Process Res Dev.

[30] Mancuso AJ, Swern D (1981). Activated dimethyl sulfoxide: useful reagents for synthesis. Synthesis..

[31] Zeiger E, Anderson B, Haworth S, Lawlor T, Mortelmans K, Speck W (1987). Salmonella mutagenicity tests: III. Results from the testing of 255 chemicals. Environmental Mutagenesis.

[32] Nikolaides N, Schipor I, Ganem B (1995). Conversion of amines to phospho esters: decyl diethyl phosphate. Org Synth.

[33] Jokelainen K, Siitonen A, Jousimies-Somer H, Nosova T, Heine R, Salaspuro M (1996). In vitro alcohol dehydrogenase-mediated acetaldehyde production by aerobic bacteria representing the normal colonic flora in man. Alcoholism..

[34] Foster RS, Fowkes A, Cayley A, Thresher A, Werner AD, Barber CG, Kocks G, Tennant RE, Williams RV, Kane S, Stalford SA (2020). The importance of expert review to clarify ambiguous situations for (Q) SAR predictions under ICH M7. Genes Environ.

[35] Piegorsch WW, Zeiger E, Hothorn L (1991). Measuring intra-assay agreement for the Ames *Salmonella* assay. Statistical methods in toxicology. Lecture notes in medical informatics.

[36] Williams RV, DeMarini DM, Stankowski LF, Escobar PA, Zeiger E, Howe J, Elespuru R, Cross KP (2019). Are all bacterial strains required by OECD mutagenicity test guideline TG471 needed?. Mutat Res.

